# Supplementary material for: Pyruvate Dehydrogenase Kinase 1 inhibition mediated oxidative phosphorylation enhancement in cartilage promotes osteoarthritis progression
Source: BMC Musculoskelet Disord. 2023 Jul 20;24:597. doi: 10.1186/s12891-023-06585-6 (PMC10357736; doi:10.1186/s12891-023-06585-6)
Supplement: Supplementary file 1 — Additional file 1. [file 12891_2023_6585_MOESM1_ESM.zip › KEGG.Gsea.1653622432464/KEGG_BETA_ALANINE_METABOLISM.html]

Details for gene set KEGG\_BETA\_ALANINE\_METABOLISM[GSEA]

|  || Dataset | 55457\_collapsed\_to\_symbols.cls.cls #OA\_versus\_normal.cls.cls #OA\_versus\_normal\_repos |
| Phenotype | cls.cls#OA\_versus\_normal\_repos |
| Upregulated in class | OA |
| GeneSet | KEGG\_BETA\_ALANINE\_METABOLISM |
| Enrichment Score (ES) | 0.36586973 |
| Normalized Enrichment Score (NES) | 1.2223696 |
| Nominal p-value | 0.20243903 |
| FDR q-value | 0.298467 |
| FWER p-Value | 1.0 |
Table: GSEA Results Summary

  

Fig 1: Enrichment plot: KEGG\_BETA\_ALANINE\_METABOLISM      
 Profile of the Running ES Score & Positions of GeneSet Members on the Rank Ordered List

  

| SYMBOL | TITLE | RANK IN GENE LIST | RANK METRIC SCORE | RUNNING ES | CORE ENRICHMENT || 1 | DPYS | dihydropyrimidinase [Source:HGNC Symbol;Acc:HGNC:3013] | 509 | 0.656 | 0.0747 | Yes |
| 2 | ALDH2 | aldehyde dehydrogenase 2 family member [Source:HGNC Symbol;Acc:HGNC:404] | 1003 | 0.517 | 0.1264 | Yes |
| 3 | ALDH3A2 | aldehyde dehydrogenase 3 family member A2 [Source:HGNC Symbol;Acc:HGNC:403] | 1263 | 0.465 | 0.1873 | Yes |
| 4 | HIBCH | 3-hydroxyisobutyryl-CoA hydrolase [Source:HGNC Symbol;Acc:HGNC:4908] | 1526 | 0.424 | 0.2408 | Yes |
| 5 | ECHS1 | "enoyl-CoA hydratase, short chain 1 [Source:HGNC Symbol;Acc:HGNC:3151]" | 1925 | 0.369 | 0.2743 | Yes |
| 6 | ALDH7A1 | aldehyde dehydrogenase 7 family member A1 [Source:HGNC Symbol;Acc:HGNC:877] | 2158 | 0.343 | 0.3160 | Yes |
| 7 | GAD1 | glutamate decarboxylase 1 [Source:HGNC Symbol;Acc:HGNC:4092] | 2534 | 0.299 | 0.3390 | Yes |
| 8 | ALDH9A1 | aldehyde dehydrogenase 9 family member A1 [Source:HGNC Symbol;Acc:HGNC:412] | 2795 | 0.270 | 0.3659 | Yes |
| 9 | DPYD | dihydropyrimidine dehydrogenase [Source:HGNC Symbol;Acc:HGNC:3012] | 3830 | 0.173 | 0.3158 | No |
| 10 | HADHA | hydroxyacyl-CoA dehydrogenase trifunctional multienzyme complex subunit alpha [Source:HGNC Symbol;Acc:HGNC:4801] | 4163 | 0.141 | 0.3146 | No |
| 11 | MLYCD | malonyl-CoA decarboxylase [Source:HGNC Symbol;Acc:HGNC:7150] | 4852 | 0.083 | 0.2757 | No |
| 12 | ACADM | acyl-CoA dehydrogenase medium chain [Source:HGNC Symbol;Acc:HGNC:89] | 4983 | 0.074 | 0.2785 | No |
| 13 | ALDH1B1 | aldehyde dehydrogenase 1 family member B1 [Source:HGNC Symbol;Acc:HGNC:407] | 6551 | -0.046 | 0.1648 | No |
| 14 | ABAT | 4-aminobutyrate aminotransferase [Source:HGNC Symbol;Acc:HGNC:23] | 6804 | -0.063 | 0.1562 | No |
| 15 | SRM | spermidine synthase [Source:HGNC Symbol;Acc:HGNC:11296] | 7735 | -0.136 | 0.1078 | No |
| 16 | EHHADH | enoyl-CoA hydratase and 3-hydroxyacyl CoA dehydrogenase [Source:HGNC Symbol;Acc:HGNC:3247] | 7968 | -0.153 | 0.1164 | No |
| 17 | SMS | spermine synthase [Source:HGNC Symbol;Acc:HGNC:11123] | 8884 | -0.223 | 0.0842 | No |
| 18 | GAD2 | glutamate decarboxylase 2 [Source:HGNC Symbol;Acc:HGNC:4093] | 9249 | -0.252 | 0.0998 | No |
| 19 | AOC3 | amine oxidase copper containing 3 [Source:HGNC Symbol;Acc:HGNC:550] | 9375 | -0.262 | 0.1357 | No |
| 20 | UPB1 | beta-ureidopropionase 1 [Source:HGNC Symbol;Acc:HGNC:16297] | 9609 | -0.284 | 0.1670 | No |
| 21 | AOC2 | amine oxidase copper containing 2 [Source:HGNC Symbol;Acc:HGNC:549] | 11550 | -0.508 | 0.1048 | No |
Table: GSEA details [plain text format]

  

Fig 2: KEGG\_BETA\_ALANINE\_METABOLISM      
 Blue-Pink O' Gram in the Space of the Analyzed GeneSet

  

Fig 3: KEGG\_BETA\_ALANINE\_METABOLISM: Random ES distribution      
 Gene set null distribution of ES for **KEGG\_BETA\_ALANINE\_METABOLISM**
